# Subsequent ipsi- and contralateral femoral fractures after intramedullary nailing of a trochanteric or subtrochanteric fracture: a cohort study on 2012 patients

**DOI:** 10.1186/s12891-022-05340-7

**Published:** 2022-04-28

**Authors:** Kirsten Marie Larsen Grønhaug, Eva Dybvik, Jan-Erik Gjertsen, Kristian Samuelsson, Bengt Östman

**Affiliations:** 1 Department of Orthopaedic Surgery, Østfold Hospital Trust, Sarpsborg, Norway; 2 Department of Clinical Medicine, University of Bergen, Bergen, Norway; 3 Norwegian Hip Fracture Register, Department of Orthopaedic Surgery, Haukeland University Hospital, Bergen, Norway; 4 Department of Orthopaedics, Institute of Clinical Sciences, Sahlgrenska Academy, University of Gothenburg, Gothenburg, Sweden; 5 Department of Orthopaedics, Sahlgrenska University Hospital, Mölndal, Sweden

**Keywords:** Subsequent femoral fracture, Intramedullary nail, Trochanteric fracture

## Abstract

**Background:**

The literature is inconclusive as to whether an intramedullary nail changes the distribution of a subsequent ipsi- or contralateral fracture of the femur. We have compared the incidence, localisation, and fracture pattern of subsequent femoral fractures after intramedullary nailing of trochanteric or subtrochanteric fractures in patients without previous implants in either femur at the time of surgery.

**Methods:**

Retrospective analysis was performed of a two-centre cohort of 2012 patients treated with a short or long intramedullary nail for the management of trochanteric or subtrochanteric fracture between January 2005 and December 2018. Subsequent presentations with ipsi- and contralateral femoral fractures were documented. Only patients with no previous femoral surgery performed, other than the index nailing were followed. Odds ratios (ORs) for subsequent femoral fracture were calculated using robust variance estimates in logistic regression.

**Results:**

The mean age of the cohort was 82.4 years and 72.1% were female. The total number of patients presenting with subsequent femoral fractures was 299 (14.9%). The number of patients presenting with subsequent ipsilateral and contralateral femoral fractures was 51 (2.5%) and 248 (12.3%) respectively (OR 5.0; CI 3.7–6.9). Twenty-six (8.7%) of all subsequent femoral fractures occured in the ipsilateral shaft, 14 (4.7%) in the ipsilateral metaphyseal area, one (0.33%) in the contralateral shaft, and three (1.0%) in the contralateral metaphysis (OR 10; CI 3.6–29).

**Conclusion:**

An intramedullary nail significantly changes the fracture pattern in the event of a second low-energy trauma, reducing the risk of subsequent proximal ipsilateral femoral fractures and increasing the risk of subsequent ipsilateral femoral fractures in the shaft and distal metaphyseal area compared with the native contralateral femur.

## Background

The incidence of a subsequent femoral fracture (Sffx) is significant among patients who have suffered an initial hip fracture [1–3]. Previous studies have stated that 2–12% of patients with a hip fracture of any type sustain a contralateral Sffx. Sffx pattern will be influenced by the presence and type of implant in situ at the time of reinjury [3–6]. Ipsilateral Sffxs appear to be less common [3]. An implant may increase the risk of some fracture types and decrease the risk of others. Without securing a native contralateral femur pre- and postoperatively, it is impossible to evaluate the true impact of the implant on Sffx.

Schröder et al. [3] reported that 92% of all Sffxs are contralateral, but without documenting any pre-existing implant in the contralateral femur at the time of surgery. The risk of an ipsilateral Sffx is higher after an initial fracture in the trochanteric, subtrochanteric or shaft region, as compared to a femoral neck fracture [6–8].

For the past several decades, fractures in the trochanteric area have been treated with either a variation of a sliding hip screw (SHS) or a short or long intramedullary nail (IMN) [9–11]. SHS is regarded as the gold standard for the stable two-fragment fractures (AO/OTA 3 1 A1) [12]. However, in the treatment of intertrochanteric (AO/OTA 3 1 A3) and subtrochanteric fractures (AO/OTA 3 2 A/B/C 1–3), the literature suggests that an IMN is a more favorable treatment option [13–17], due to the shorter lever arm and the reduced potential for medialization the nail device provides. Despite the biomechanical benefits, IMNs have been associated with an increased risk of ipsilateral Sffx, although this appears to be less frequent with contemporary nail designs [18, 19]. The choice of implant may influence the incidence, localisation and morphology of a Sffx [20, 21]. In a recent retrospective study, no differences could be found when comparing the incidence of a contralateral Sffx after an initial trochanteric fracture treated with either an IMN or an SHS [22].

This study aimed to investigate how an IMN affects the incidence, pattern and localisation of Sffxs in patients treated for a trochanteric or subtrochanteric fracture with documented normal femora without implants or sequelae after previous surgery in either femur. A comparison between an operated femur and a persistently native contralateral femur will inevitably require exclusion of patients with any femoral implant initially and consecutive censoring of patients having subsequent implant surgery in either femur. The chosen design intends to reduce the influence of individual characteristics difficult to account for, such as risk behavior, drug or alcohol abuse, fall tendency, and comorbidity.

## Methods

### Study design

In this retrospective cohort study all patients with a trochanteric or subtrochanteric femoral fracture treated with a short or long IMN at Østfold Hospital Trust between 2005 and 2018 (*n* = 2525) were eligible for inclusion. To study the true impact of an IMN on the distribution of Sffxs after intramedullary nailing of trochanteric and subtrochanteric fractures, the pre-operative status in both femora was investigated. Comparison of the operated femur with the contralateral femur was only continued as long as no major surgery was performed on either femur. The occurrence of and time to a new admission due to a Sffx was registered, yielding total exposure time.

### Sources of data

Patients were identified by searching the hospital databases using the International Statistical Classification of Diseases and Related Health Problems (ICD10) and the NOMESKO Classification of Surgical Procedures (NCSP) codes. Patients with ICD10 codes S72.1 (per- or intertrochanteric fractures) or S72.2 (subtrochanteric fractures) and NCSP codes NFJ51 or NFJ52 (intramedullary nailing of trochanteric and subtrochanteric fractures respectively) were identified. Electronic health records and X-ray images were reviewed. Handwritten documentation provided by the surgeons postoperatively was reviewed and compared with the electronic health records. The American Society of Anesthesiologists Physical Status scoring system (ASA score) was used to assess the overall health status of the patient.

### Identification of cohort

Pre- and postoperative X-ray images, as well as all follow-up X-ray images taken of the proximal femur and the pelvis, were examined to identify, classify and localise index fractures and Sffxs. Fracture type was registered according to the AO/OTA classification system [23]. Pelvic X-ray images visualising the contralateral hip and all follow-up X-ray images were included for all patients to identify any pre-existing implant, added implant, or sequelae in either proximal femur.

### Exclusion process

**Fig. 1:**
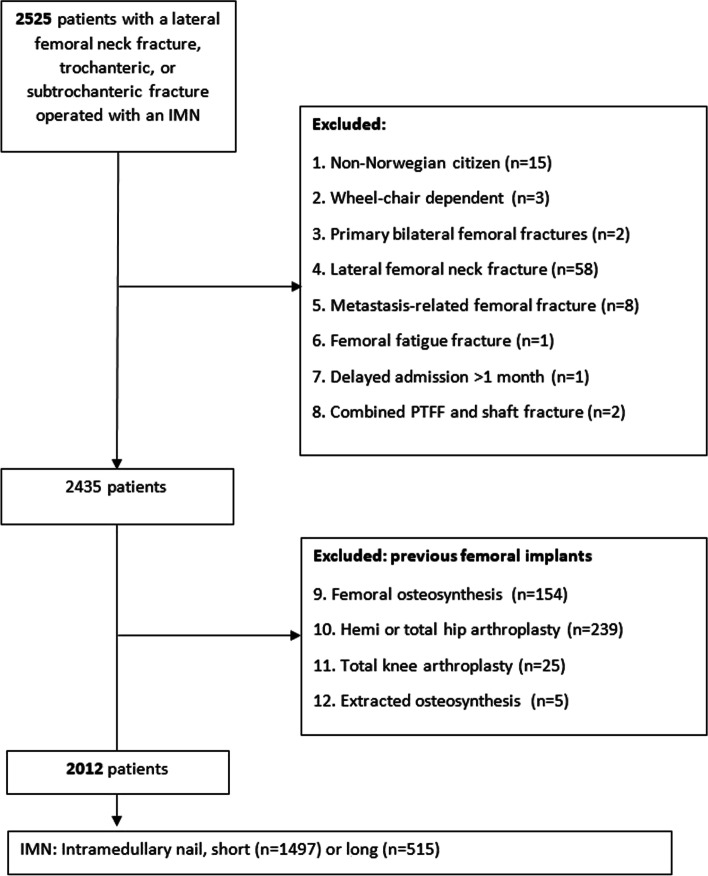
Flow chart of exclusion process

Patients < 60 years of age, non-Norwegian citizens, patients with the primary care episode at a non-orthopaedic department, a pre-existing implant in either femur, high-energy trauma, multiple simultaneous fractures in the lower extremities or pathologic fracture (other than osteoporosis) were excluded from the study. Finally, 2012 patients met the inclusion criteria. The exclusion process is illustrated in Fig. 1.

### Censoring

The following endpoints were registered: ipsi- and contralateral Sffxs (medial or lateral femoral neck fractures, trochanteric avulsions, per- or subtrochanteric fractures, femoral shaft fractures, distal femoral fractures), infection, cut-out, failure of osteosynthesis, non-union, mal-union, local pain, local hematoma, lag screw complications leading to surgery and surgery involving a non-fracture-related implant in either femur (total or hemi-arthroplasty in the hip or knee).

### Validation

Data obtained from electronic health records were validated using a comparison with data from the Norwegian Hip Fracture Register (NHFR) [24]. A total of 329 (16.4%) patients who underwent surgery were not registered in the NHFR while 42 (2.1%) patients were registered in the NHFR but not in the local electronic health records. All patients were included.

### Ethics

The study was approved by the Norwegian Regional Ethics Committee (REC South-East). The STROBE (STrengthening the Reporting of OBservational studies in Epidemiology) guidelines were followed [25].

### Statistical analysis

The data was summarised using frequencies and percentages for categorical variables. The mean, standard deviation (SD) and range were calculated for continuous variables. The mean time to subsequent fracture, range and 95% confidence interval (CI) were calculated. The alpha value was set at 0.05. The odds ratio (OR) was calculated using robust variance estimates in logistic regression. The follow-up time was calculated from the primary operation until a new fracture-related operation on the ipsilateral side, fracture-related surgery on the contralateral side, other operation not related to a fracture, death, or 31 January 2020, whichever came first. The patients who reached any of the endpoints were censored along the way. The statistical analyses were performed using IBM-SPSS Statistics (Version 24.0, IBM, Armonk, NY, USA), the R, version 3.4.0, statistical package (http://www.R-project.org) and Stata/SE (Version 16.0, StataCorp LLC, College Station, TX, USA).

## Results

Table 1 presents the baseline characteristics of the 2012 patients included. The mean age was 82.4 years and 72.1% were female. Out of the 2012 patients, 1890 (93.9%) were classified as ASA 2 or ASA 3 prior to surgery. The index fracture was pertrochanteric in 1959 (97.4%) cases. Laterality was near symmetrically distributed (right: 48.8%, left: 51.2%). The mean exposure time was 1031 days (SD 1036).

**Table 1:** Baseline data

Characteristics	
**Total n**	**2012**
Gender, n (%)	
Male	562 (27.9)
Female	1450 (72.1)
Mean age, (SD)(range)	82.4 (8.5) (60–103)
ASA, n (%)	
1	31 (1.5)
2	829 (41.2)
3	1059 (52.6)
4	89 (4.4)
Missing	4 (0.20)
Type of fracture, n (%)	
Pertrochanteric	1959 (97.4)
Subtrochanteric	53 (2.6)
Laterality, n (%)	
Right	981 (48.8)
Left	1031 (51.2)
Mean exposure in days, (SD)(range)	1031 (1036) (0–5379)

A Short Gamma3 Intramedullary Nail (Stryker Corporation, Kalamazoo, Michigan, USA) alone accounted for 55.8% of the implants used, followed by long Gamma3 (21.1%), (Table 2). The remaining implants were divided between the GammaT nail, the TRIGEN InterTan Nail (Smith and Nephew, Hertfordshire, UK), the AFFIXUS Hip Fracture Nail (Zimmer Biomet, Indiana, USA) and, in a single case, the TRIGEN Trochanteric Nail (Smith and Nephew, Hertfordshire, UK). A long nail was used in 515 (25.6%) cases.

**Table 2:** Type of implant

Implant	Type	Number (% of total)	With distal locking (%)
Short nail	**Total**	**1497 (74.4)**	**1326 (88.6)**
	GammaT	198 (9.8)	129 (65.2)
	Gamma3	1122 (55.8)	1020 (90.9)
	InterTan	174 (8.6)	174 (100.0)
	Affixus	3 (0.15)	3 (100.0)
Long nail	**Total**	**515 (25.6)**	**513 (99.6)**
	GammaT	32 (1.6)	31 (96.9)
	Gamma3	424 (21.1)	423 (99.8)
	InterTan	57 (2.8)	57 (100.0)
	Affixus	1 (0.05)	1 (100.0)
	Trigen	1 (0.05)	1 (100.0)

[Stryker GammaT Intramedullary Nail TM (2005–2007), Stryker Gamma3 Intramedullary Nail TM (2007–2017), Smith and Nephew TRIGEN Trochanteric Nail TM (2015), Smith and Nephew TRIGEN InterTan Nail TM (2016–2017), Zimmer Biomet AFFIXUS Hip Fracture Nail TM (2017)].

**Fig. 2:**
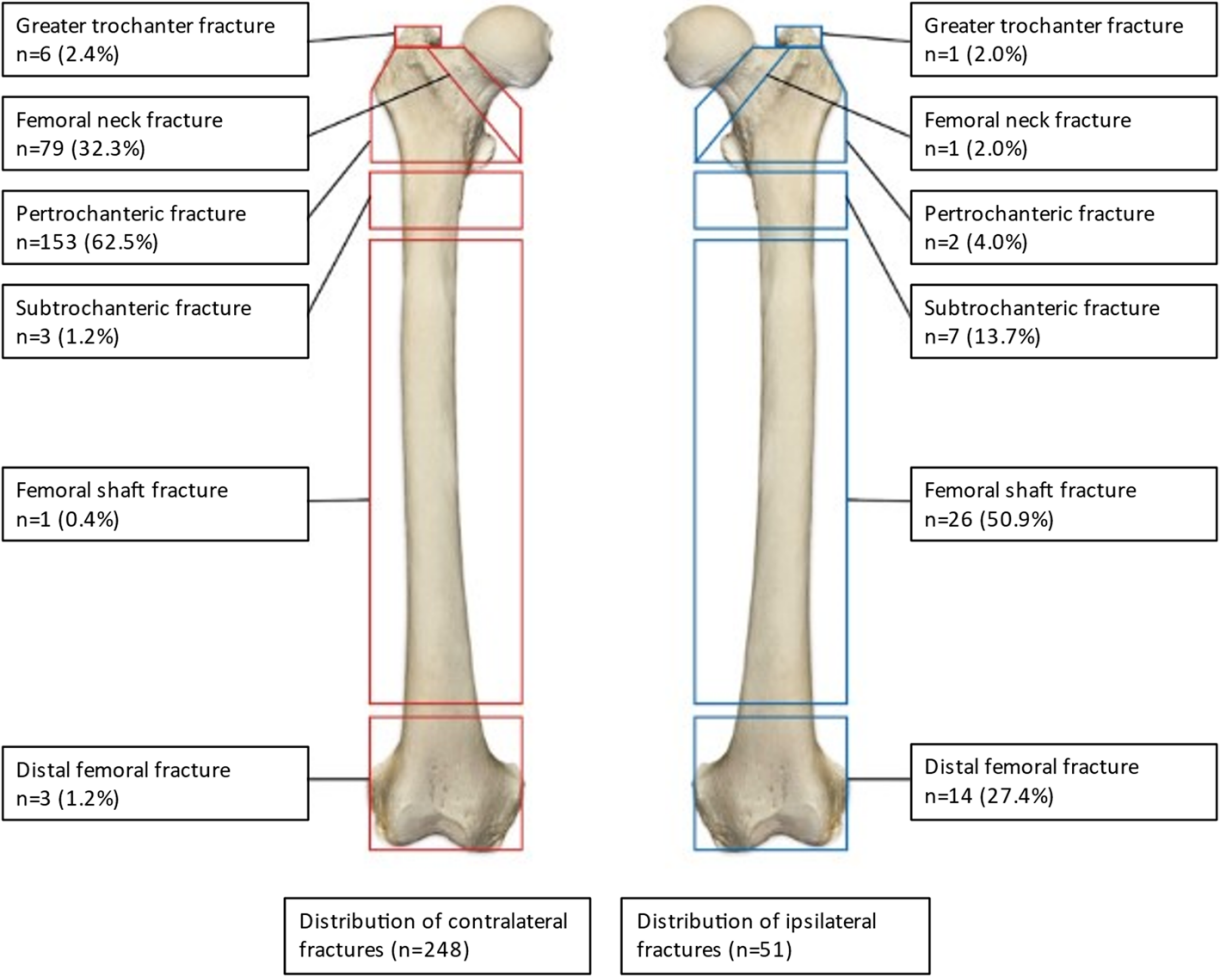
Distribution of subsequent femoral fractures

The total number of Sffxs was 299 (14.9%). Ipsilateral Sffxs occurred in 51 (2.5%) and contralateral in 248 (12.3%) of all patients (OR 5.0; CI 3.7–6.9, *p* < 0.001). The distribution of Sffxs is illustrated in Fig. 2. A significantly higher rate of ipsilateral Sffxs in the shaft and distal metaphyseal area was detected compared with the corresponding types of contralateral Sffxs (OR 10; CI3.6–29, *p* < 0.001). Ipsilateral Sffxs in the shaft accounted for 8.7% (*n* = 26) and in the metaphyseal area 4.7% (*n* = 14) of all Sffxs. The corresponding figures for contralateral Sffxs was 0.33% (*n* = 1) and 1.0% (*n* = 3) respectively. In the contralateral femur, 238 (79.6%) Sffxs occurred in the femoral neck and per- and subtrochanteric area, compared to 10 (3.3%) in the ipsilateral femur.

Of 1497 patients treated with a short nail, six (0.4%) sustained an ipsilateral subtrochanteric fracture, 23 (1.5%) sustained a shaft fracture, and 10 (0.6%) sustained a Sffx in the metaphyseal area. Of 515 patients treated with a long nail, one (0.19%) sustained an ipsilateral subtrochanteric fracture, three (0.6%) sustained a shaft fracture, and five (1.0%) sustained a Sffx in the metaphyseal area. Three (0.6%) ipsilateral Sffxs occurred in the proximal part of the femur and only in patients treated with a short nail.

Patients reaching any endpoint were censored along the way. Table 3 shows the distribution of all endpoints. Non-fracture-related complications leading to reoperation and non-fracture-related operations in either femur reached a total of 123 (6.1%) censored patients. Cut-out and other lag screw complications were the most prevalent non-fracture complications leading to reoperation in 40 (2.0%) and 17 (0.8%) patients respectively. When ruling out contra- and ipsilateral coxarthrosis, 118 (5.9%) complications directly related to the primary operation were identified. A total of 1226 (60.9%) patients died without experiencing a Sffx or any non-fracture complications.

**Table 3:** Endpoints of study

Endpoint		N	%
Subsequent femoral fracture	**Total**	**299**	**14.9**
	Ipsilateral	51	2.5
	Contralateral	248	12.3
Other complications requiring surgery	**Total**	**118**	**5.9**
	Infection	16	0.8
	Cut-out	40	2.0
	Failure of osteosynthesis	5	0.25
	Non-union	21	1.0
	Mal-union	2	0.10
	Local pain	17	0.8
	Screw complications	17	0.8
Non-fracture related surgery	Coxarthrosis –THA	**17**	**0.8**
Death		**1226**	**60.9**

*THA* Total hip arthroplasty.

The number of patients left at risk nearly halved every two years. Table 4 shows the distribution of endpoints in two-year intervals.

**Table 4:** Risk of ipsi- and contralateral fracture. Censored data are substracted consecutively

	Years					
Endpoint	0–2	2–4	4–6	6–8	8–10	> 10
Number left at risk	2012	1029	566	283	137	53
Death	714	258	152	59	24	16
Non-fracure rel. Reoperation	104	9	3	2	1	1
Ipsilateral Sffx^a^	35	9	5	1	0	1
Contralateral Sffx^a^	122	68	27	18	10	3
End of follow-up	8	119	96	66	49	32

^a^ Subsequent femoral fracture

## Discussion

This retrospective study of 2012 patients treated with an intramedullary nail for a pertrochanteric or subtrochanteric fracture investigated the occurrence of subsequent femoral fractures. Patients with coexisting implants or fracture sequelae in either femur were excluded to assess the impact of the IMN alone. The total incidence of a Sffx was five times lower on the ipsilateral than the contralateral side following surgery with an IMN. However, the study demonstrated a tenfold increase in the risk of ipsilateral femoral shaft and distal fractures after receiving an IMN for a trochanteric or subtrochanteric fracture, compared with a contralateral side without implants or fracture sequelae.

To the best of our knowledge, no previous study has compared the impact of IMN in the treatment of trochanteric or subtrochanteric fractures in elderly patients with no previous implant in either femur at the time of surgery. The incidence of peri-implant femoral fractures distal to an IMN has been described [20, 26]. Although the incidence has decreased with the evolution of new generations of IMNs, peri-implant fractures still represent a challenge to the orthopaedic surgeon. The overall lower risk of an ipsilateral Sffx, as well as the increased incidence of those ipsilateral Sffxs occurring in the shaft and distal metaphyseal area, are consistent with the existing literature [7, 20, 27–30]. However, previous studies have not specified the presence or absence of other implants or sequelae in either femur prior to the index fracture. Bögl et al. concluded that there was a reduced risk of subsequent hip fracture with the use of IMNs with femoral neck protection in the treatment of low energy femoral shaft fractures in a retrospective study of 897 patients, but discuss that national register data are incomplete regarding laterality [31]. Schröder et al. did not review x-ray images until a subsequent femoral fracture was present [3]. By reviewing all x-ray images to exclude patients with pre-existing implants in the present study we were able to further assess the impact of the IMN.

All the Sffxs in our study occurred as a result of low-energy trauma, as high-energy injured patients were excluded. Biomechanical studies and clinical experience suggest that the incidence of peri-implant fractures is affected by the modulus of elasticity of the implant [32]. The increased incidence of ipsilateral versus contralateral Sffxs in the shaft and distal metaphyseal area following the implantation of an IMN in this study may indicate a redistribution of forces in the event of a second trauma to the femur – protecting the proximal femur but yielding an increased risk of fractures distal to the nail. However, the protective effect of the IMN with regard to an ipsilateral proximal Sffx is far greater than the increased risk of an ipsilateral peri-implant fracture. Extracting an IMN increases the risk of sustaining an ipsilateral trochanteric fracture substantially compared with the risk of sustaining an ipsilateral peri-implant fracture with the implant in place.

The increased incidence of low-energy Sffx distal to an implanted IMN, as seen in this study, may be a result of morphologic changes in the adjacent bone secondary to the implant. The rigidity of the chosen implant causes the implant to bear the majority of the load [33], resulting in stress-shielding and bone resorption around the implant over time [34–36]. The stress-shielding effect causes localised osteopenia as well as inactivity osteopenia and is likely to contribute to the increased risk of ipsilateral Sffxs in the shaft and distal metaphyseal area demonstrated in this study. Previous studies have confirmed the rapid reduction of bone mineral density (BMD) following postoperative immobilisation [30, 37–39]. Postoperative restraints reduce BMD in the entire femur and may contribute to the increased incidence of ipsilateral Sffxs in the shaft and distal metaphyseal area. Furthermore, previous studies rate impaired balance and an increased risk of falling as even more important risk factors for hip fractures than low BMD [40–42].

The overall risk of an Sffx is significant after any low-energy index fracture, as demonstrated in the work of Center [43] and Kanis [2]. In a population-based Finnish study, the risk of an Sffx was significantly higher than the risk of a primary hip fracture during the first twelve to fifteen months postoperatively [44]. The population most vulnerable to fractures in the trochanteric area typically display sarcopenia, as defined by the European Working Group on Sarcopenia in Older Persons (EWGSOP): low muscle mass with low muscle strength or low physical performance [45]. This deteriorates further after the index hip fracture [46–48]. Reduced activity, mobility, pain, fear, and avoidance enhance sarcopenia, thereby increasing the risk of falling [38].

### Strengths and weaknesses

The strengths of this study include the large sample size, the long follow-up, the exclusion of patients with pre-existing implants or sequelae in either femur prior to the index nailing and validation by comparison with data from NHFR. The discrepancy identified during the validation process may be due to surgeons forgetting to report to the NHFR, missing data in received forms, forms lost in transit and patients with comorbidities warranting the complete care episode at a non-orthopedic department.

The study has several limitations. Due to the retrospective design, no information was available on BMD or fall pattern before or after the index operation. However, by only including patients without previous implants in either femur, we assumed a similar BMD in both femurs at the time of index fracture. Although fracture type may influence the risk of Sffx [3, 6–8], subanalyses of index fracture type or stability were not conducted, as ipsilateral Sffxs are relatively uncommon. As this study aimed at comparing a native femur, operated with an IMN, with a persistently native contralateral femur, we excluded patients with any femoral implant initially, and even censored patients having subsequent non-fracture related surgery in either femur later on. Such a study design is advantageous in view of our aim but significantly limits the ability to compare the influence of nail length on the distribution of ipsilateral Sffxs. This study only included patients treated with IMNs, and it cannot be ascertained whether the incidence and fracture pattern of Sffxs found in this study are specific to IMNs or apply to other femoral implants as well, such as sliding hip screws or arthroplasty femoral stems.

## Conclusion

An IMN significantly changes the fracture pattern in the event of a second low-energy trauma by reducing the risk of proximal Sffxs but increasing the risk of Sffxs in the shaft and distal metaphyseal area compared with the native contralateral femur. The overall risk of Sffx is substantially higher in the contralateral, native femur indicating the protective effect of the IMN with regard to a proximal ipsilateral Sffx is much higher than the increased risk of sustaining an ipsilateral peri-implant fracture.

Given the serious nature of peri-implant fractures, further biomechanical studies and clinical research to investigate why IMNs lead to increased fracture rates distal to the implant are called for, as well as similar studies investigating the risk of Sffx after other intra- and extramedullary implants in the treatment of proximal femoral fractures.

## Data Availability

The regulations of the Norwegian Data Protection Authority and the Norwegian personal protection laws prohibit the publication of the complete dataset. The data that support the findings of this study are available from the Norwegian Hip Fracture Register but restrictions apply to the availability of these data, which were used under license for the current study, and so are not publicly available. Data are however available from the authors upon request and with permission of the Norwegian Hip Fracture Register.

## Abbreviations

AO/OTA: Arbeitsgemeinscahft für Osteosynthesefragen/Orthopaedic Trauma Association
ASA: The American Society of Anesthesiologists
BMD: Bone Mineral Density
EWGSOP: European Working Group on Sarcopenia in Older Persons
ICD-10: International Statistical Classification of Diseases and Related Health Problems
IMN: Intramedullary Nail
NHFR: Norwegian Hip Fracture Register
NCSP: NOMESKO Classification of Surgical Procedures
REC: Norwegian Regional Ethics Committee
Sffx: Subsequent femoral fracture
SHS: Sliding Hip Screw
STROBE: STrengthening the Reporting of OBservational studies in Epidemiology
